# A Non-Mammalian Type Opsin 5 Functions Dually in the Photoreceptive and Non-Photoreceptive Organs of Birds

**DOI:** 10.1371/journal.pone.0031534

**Published:** 2012-02-14

**Authors:** Hideyo Ohuchi, Takahiro Yamashita, Sayuri Tomonari, Sari Fujita-Yanagibayashi, Kazumi Sakai, Sumihare Noji, Yoshinori Shichida

**Affiliations:** 1 Department of Life Systems, Institute of Technology and Science, University of Tokushima Graduate School, Tokushima, Japan; 2 Department of Biophysics, Graduate School of Science, Kyoto University, Kyoto, Japan; University of Washington, United States of America

## Abstract

A mammalian type opsin 5 (neuropsin) is a recently identified ultraviolet (UV)-sensitive pigment of the retina and other photosensitive organs in birds. Two other opsin 5-related molecules have been found in the genomes of non-mammalian vertebrates. However, their functions have not been examined as yet. Here, we identify the molecular properties of a second avian opsin 5, cOpn5L2 (chicken opsin 5-like 2), and its localization in the post-hatch chicken. Spectrophotometric analysis and radionucleotide-binding assay have revealed that cOpn5L2 is a UV-sensitive bistable pigment that couples with the Gi subtype of guanine nucleotide-binding protein (G protein). As a bistable pigment, it also shows the direct binding ability to agonist all-*trans*-retinal to activate G protein. The absorption maxima of UV-light-absorbing and visible light-absorbing forms were 350 and 521 nm, respectively. Expression analysis showed relatively high expression of *cOpn5L2* mRNA in the adrenal gland, which is not photoreceptive but an endocrine organ, while lower expression was found in the brain and retina. At the protein level, cOpn5L2 immunoreactive cells were present in the chromaffin cells of the adrenal gland. In the brain, cOpn5L2 immunoreactive cells were found in the paraventricular and supraoptic nuclei of the anterior hypothalamus, known for photoreceptive deep brain areas. In the retina, cOpn5L2 protein was localized to subsets of cells in the ganglion cell layer and the inner nuclear layer. These results suggest that the non-mammalian type opsin 5 (Opn5L2) functions as a second UV sensor in the photoreceptive organs, while it might function as chemosensor using its direct binding ability to agonist all-*trans*-retinal in non-photoreceptive organs such as the adrenal gland of birds.

## Introduction

Light has been exploited for information by organisms throughout the evolution of photoreceptors and, ultimately, eyes and other photoreceptive organs in animals. The transduction of photons into cellular signals uses seven transmembrane-spanning opsin proteins that combine with a vitamin A-derived, nonprotein retinal chromophore. Opsins, which control sensitivity to light of different wavelengths, have evolved into at least seven distinct families (reviewed in [1], [2], [3]). These families transduce light using trimetric G protein-coupled mechanisms and consist of 1) invertebrate visual opsins and vertebrate melanopsin (opsin 4) that couple with Gq-type G proteins, 2) vertebrate visual opsins, four kinds of non-visual opsins (parietopsins, pinopsins, parapinopsins, and vertebrate ancient opsins), encephalopsins and teleost multiple tissue opsins (opsin 3), which mainly couple with Gi group G proteins including Gt, 3) Go-coupled opsins found in scallops and amphioxus, 4) Gs-coupled opsins found in jellyfish, 5) neuropsins or opsin 5, 6) peropsins, and 7) the photoisomerase group.

The opsin 5 (Opn5) group was first identified in the eye, brain, testis and spinal cord in mice and humans [4]. Although Opn5 is closely related phylogenetically to retinal photoisomerases, the molecular properties of a chicken homolog of Opn5, chicken Opn5, mammalian type (cOpn5m), have been recently elucidated as an ultraviolet (UV)-sensitive bistable pigment that couples with the Gi subtype of G proteins [5]. In addition, this mammalian type Opn5 was hypothesized to mediate hypothalamic photoreception for seasonal testicular growth in birds [6]. On the other hand, it was reported that there are at least three *Opn5*-like genes in the chicken, and two of them are only found in non-mammalian vertebrates, such as birds and fish [7]. However, their molecular properties and functions, with the exception of cOpn5m, have not been characterized. Since the deduced amino acid sequence of the two other *Opn5*-related genes showed relatively low sequence identity/similarity compared with the mammalian type Opn5, it is crucial to identify the molecular properties and distribution in the retina, brain, and other organs, and determine whether these three Opn5-related molecules functionally comprise the same Opn5 subfamily.

To test this, here we determined the absorption spectrum, the retinal configurations, and the G protein activation ability of a chicken Opn5-like 2, cOpn5L2, reconstituted in cultured cells. We further examined the expression pattern of cOpn5L2 in various tissues at the mRNA and protein levels. Our results show that cOpn5L2 is a member of the Opn5 subfamily based on the molecular properties, while it has a distinct expression pattern from that of the previously-identified mammalian type Opn5, suggesting its dual roles in the photoreceptive and non-photoreceptive organs of birds.

## Results

### Molecular properties of cOpn5L2

**Figure 1:**
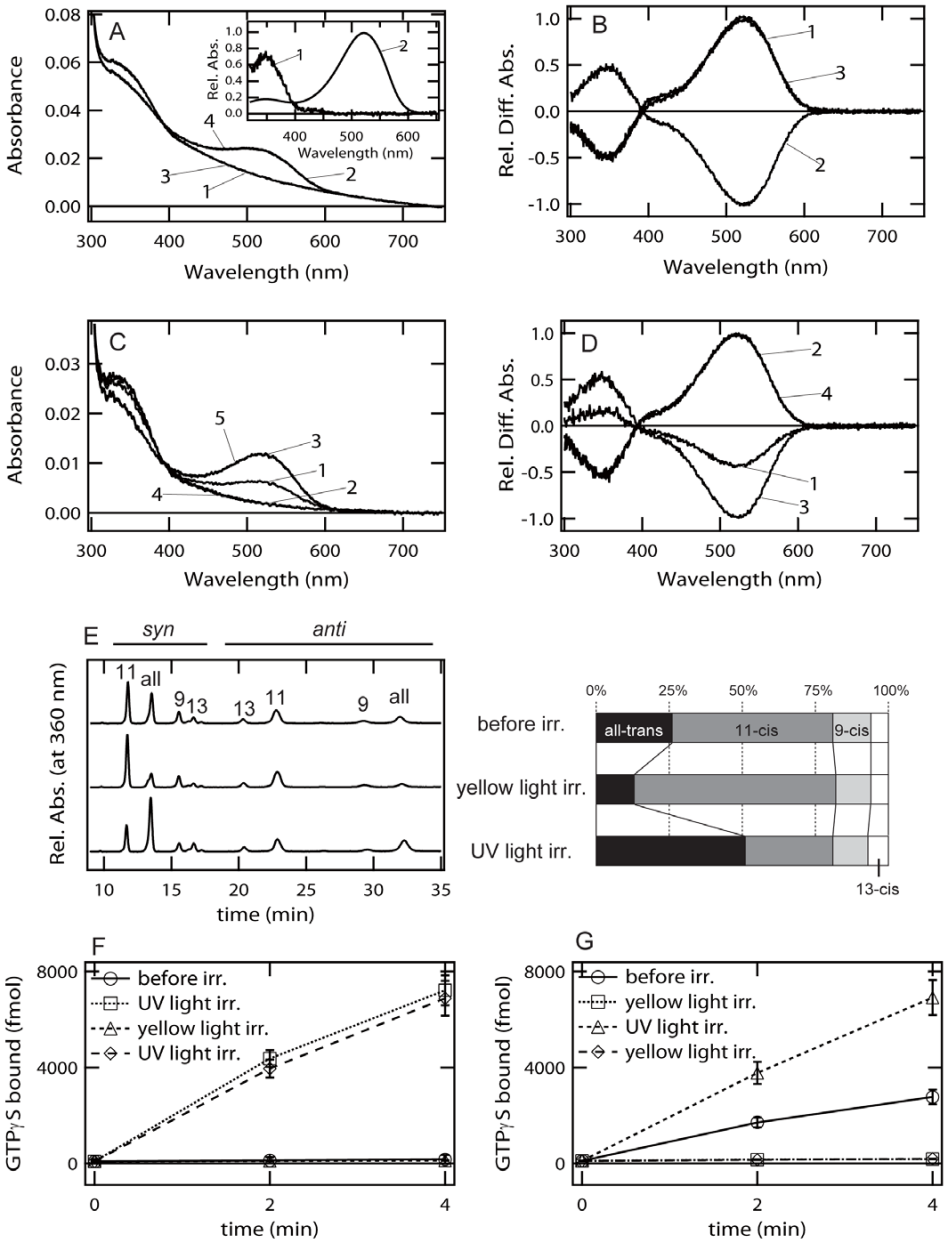
Molecular properties of cOpn5L2. Absorption spectra, retinal configurations and G protein activation of cOpn5L2. ***A***, Absorption spectra of cOpn5L2 purified after incubation with 11-*cis*-retinal. Spectra were recorded in the dark (curve 1), after UV light irradiation (curve 2), after subsequent yellow light (>500 nm) irradiation (curve 3), and after UV light re-irradiation (curve 4). (inset) The calculated absorption spectra of cOpn5L2 in the dark (curve 1) and after UV light irradiation (curve 2). The calculation procedures are described in the text. ***B***, Spectral changes caused by UV light irradiation (curve 1), subsequent yellow light irradiation (curve 2), and UV light re-irradiation (curve 3). ***C***, Absorption spectra of cOpn5L2 purified after incubation with all-*trans*-retinal. Spectra were recorded in the dark (curve 1), after yellow light irradiation (curve 2), after subsequent UV light irradiation (curve 3), after yellow light re-irradiation (curve 4), and after UV light re-irradiation (curve 5). ***D***, Spectral changes caused by yellow light irradiation (curve 1), subsequent UV light irradiation (curve 2), yellow light re-irradiation (curve 3), and UV light re-irradiation (curve 4). ***E***, Retinal configurations in cOpn5L2 purified after incubation with all-*trans*-retinal. (left-hand panel) The retinal isomers before irradiation, after yellow light irradiation, and after subsequent UV light irradiation were analyzed with HPLC after extraction of the chromophore as retinal oximes (syn and anti forms of 9-*cis*-, 11-*cis*-, 13-*cis*-, and all-*trans*-retinal oximes). (right-hand panel) Isomeric compositions of retinal before and after light irradiation of cOpn5L2. ***F***, Gi-type G protein activation ability by cOpn5L2 purified after incubation with 11-*cis*-retinal. The time-dependent change of the activity was measured in the dark (open circle), after UV light irradiation (open square), after subsequent yellow light irradiation (open triangle), and after UV light re-irradiation (open diamond). ***G***, The Gi activation ability of cOpn5L2 purified after incubation with all-*trans*-retinal. The activity was measured in the dark (open circle), after yellow light irradiation (open square), after subsequent UV light irradiation (open triangle), and after yellow light re-irradiation (open diamond). G protein activation assay in (F) and (G) was performed at 0°C, and data are presented as the means ± S.D. of three independent experiments.

To investigate the molecular properties of cOpn5L2, we purified recombinant cOpn5L2 expressed in cultured cells. Figure 1A showed the absorption spectrum and the light-dependent changes of cOpn5L2 reconstituted with 11-*cis-*retinal. UV light irradiation of the pigment resulted in a shift of the spectrum into the visible region. Yellow light irradiation subsequently caused the formation of a pigment having an identical spectrum with the original one. Re-irradiation of the pigment with UV and yellow light led to identical spectrum changes, which was confirmed by the difference spectra shown in Figure 1B. Moreover, we prepared cOpn5L2 purified after incubation with all-*trans*-retinal (Figure 1C). The absorption spectrum of this pigment had two peaks; one was in the visible region, and the other was in UV region. Irradiation with yellow light on the pigment induced the formation of a pigment having an absorption maximum in the UV region. A simultaneous increase and decrease of the absorption at the visible and UV region was repeatedly observed by subsequent UV and visible light irradiations (Figure 1D). These results showed that UV and visible light-absorbing forms were inter-convertible by light irradiation. The calculated absorption spectra indicated that the absorption maxima of UV and visible light-absorbing forms are 350 nm and 521 nm, respectively (Figure 1A, inset).

We next analyzed the retinal configurations of these two forms (Figure 1E). The pigment that was purified contained a large amount of 11-*cis*-retinal after incubation with all-*trans*-retinal. This was most likely because the binding affinity of cOpn5L2 to 11-*cis*-retinal is greater than that to all-*trans*-retinal, so that 11-*cis*-retinal formed from the thermal isomerization of the all-*trans*-form in the culture medium preferentially bound to cOpn5L2. Irradiation with yellow light on the pigment increased the amount of the 11-*cis*-form and decreased the amount of the all-*trans*-form. Subsequent irradiation with UV light caused an opposite shift of retinal isomers. These results showed that UV and visible light-absorbing forms contain 11-*cis*- and all-*trans*-retinals, respectively. Moreover, these results are consistent with the results obtained from cOpn5m [5].

Our previous report revealed that cOpn5m can couple with Gi-type G proteins [5]. A sequence comparison between cOpn5m and cOpn5L2 indicated that the “glutamic/aspartic acid (E/D)-arginine (R)-tyrosine (Y)” motif in helix III, which is well-conserved among many G protein-coupled receptors (GPCRs), is maintained in cOpn5m but changed into an “isoleucine (I)-R-phenylalanine (F)” triad in the cOpn5L2 opsin [7]. Thus, we analyzed whether cOpn5L2 had efficient G protein activation ability. The pigment reconstituted with 11-*cis*-retinal was able to markedly activate Gi in a UV light-dependent manner, and the activity was suppressed by subsequent yellow light irradiation (Figure 1F). Therefore, cOpn5L2 also functions as a Gi-coupled GPCR, and the visible light-absorbing form that has all-*trans*-retinal is in an active state. In the case of the pigment incubated with all-*trans*-retinal, the dark state had detectable activity (Figure 1G). Sequential yellow light and UV light irradiation resulted in a decrease and increase of the activity, as observed in the pigment reconstituted with 11-*cis*-retinal. In consideration of the results from the spectrum and the retinal configuration of the pigment incubated with all-*trans*-retinal, all-*trans*-retinal most likely incorporates directly into cOpn5L2, which results in induction of basal constitutive activity.

### Localization of cOpnL2 in post-hatching chicken neural and endocrine tissues

**Figure 2:**
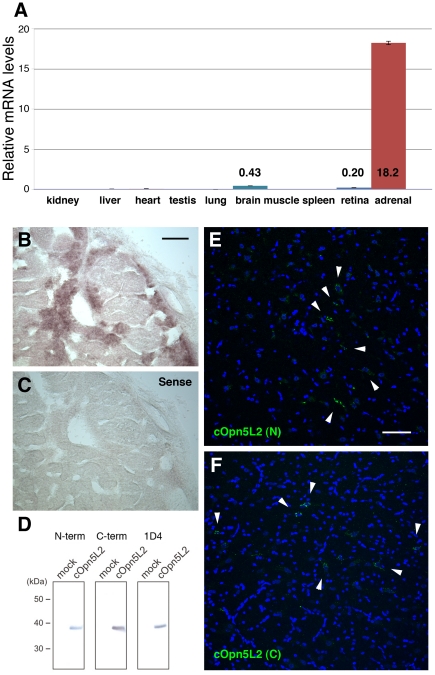
The expression pattern of cOpn5L2. Revealed by quantitative PCR (A), in situ hybridization (B, C), western blot analysis (D), and immunohistochemistry (E, F). ***A***, Quantitative PCR analysis of ten tissues (as shown) from post-hatching chick (2 weeks). *cOpn5m* mRNA level in retina is referred to as 1. ***B***, ***C***, In situ hybridization of the chick adrenal gland at post-hatching day 30 (P30). A sense probe for *cOpn5L2* shows no staining in the consecutive section (C). ***D***, (Left and middle) Western blot analysis using a cOpn5L2 (N-term) or cOpn5L2 (C-term) antibody. Cell lysates of mock or cOpn5L2-expressing cells were loaded. (Right) cOpn5L2 cDNA is tagged with C-terminal amino acids of bovine rhodopsin and its expression was confirmed using an anti-bovine rhodopsin antibody (rho1D4). ***E***, ***F***, Immunohistochemistory of the brain using cOpn5L2 (N-term) (E) or cOpn5L2 (C-term) (F) antibody. The lateral hypothalamic area (P10) is shown. Nuclei are stained with DAPI (blue). cOpn5L2 immunoreactive (IR) cells are shown in green (arrowheads). Scale bars, 100 µm (B, C), and 50 µm (E, F).

As a first step for elucidating the function of cOpn5L2, we examined relative mRNA levels of *cOpn5L2* in a panel of ten chicken tissues by quantitative polymerase chain reaction (PCR). Since relatively higher expression was observed in the post-hatching chick adrenal glands, brain, and retina (Figure 2A), we focused on characterizing the *cOpn5L2*-expressing cells in these three tissues. Standard in situ hybridization revealed that *cOpn5L2* is expressed in part of the adrenal glands of the post-hatching chick (Figure 2B, C). In contrast, we could not detect *cOpn5L2* mRNA in the post-hatching retina by standard in situ hybridization with digoxigenin-labeled probes (not shown), which indicates lower amount of mRNA expression than the sensitivity of in situ hybridization. We then raised specific antibodies against peptides corresponding to the N-terminal or C-terminal region of cOpn5L2. We found that both antibodies were specific to cOpn5L2, with anti-cOpn5L2 (N) or anti-cOpn5L2 (C) only identifying cOpn5L2-transfected cells, as shown by western blot analysis (Figure 2D). We compared the immunoreactivity of the two antibodies and did not detect noticeable differences in their staining pattern (Figure 2E, F). Since the anti-cOpn5L2 (C) antibody exhibited stronger immnunoreactivity with lower background than the anti-cOpn5L (N) antibody, we used the cOpn5L2 (C) antibody for subsequent immunostaining experiments.

### Localization of cOpn5L2 in the adrenal glands

**Figure 3:**
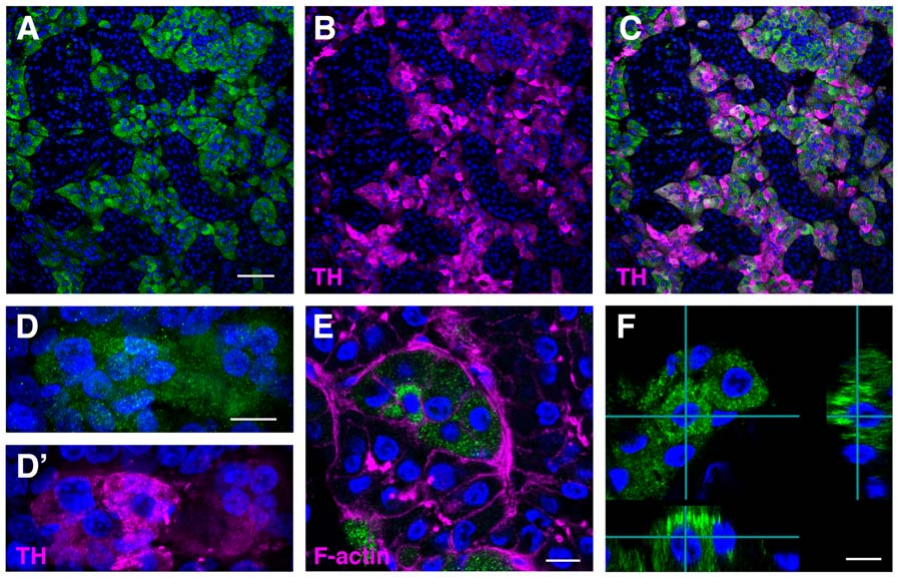
Immunohistochemistry of the adrenal gland. cOpn5L2 immunoreactivity is shown in green, cell nuclei in blue, and TH (B, C, D′) or F-actin (E) in magenta. ***A–C***, cOpn5L2 IR cells are localized to the TH-producing cells in the adrenal gland at P15. ***D***, ***D′***, High magnification of the adrenal medullary cells at P30. ***E***, to reveal the epithelial cell shape, filamentous actin is visualized by staining with rhodamine-phalloidin. cOpn5L2-immunoreactivity is observed in the cytoplasm. ***F***, The xz and yz views of a selected point in the image are shown, supporting the cOpn5L2 localization in the cytoplasm. Scale bars, 50 µm (A–C), 10 µm (D, D′, E), and 7.5 µm (F).

As mentioned above, *cOpn5L2* mRNA was detected in a portion of the adrenal glands (Figure 2B). Immunohistochemical studies also showed that cOpn5L2 protein was localized to a portion of the adrenal glands (Figure 3A). The adrenal glands consist of two distinct cell lineages; the adrenal cortex, which is derived from the mesoderm, similar to the urogenital system, and produces steroids, and the medulla, which is derived from the neural crest, similar to the sympathetic nervous system, and produces catecholamines. However, in avian adrenal glands, the cortical and medullary tissues are intermingled throughout the gland, which is different from that of mammals. To determine whether cOpn5L2 immunoreactive (IR) cells are cortical or medullary, double immunostaining was performed using the anti-cOpn5L2 antibody together with the anti-tyrosine hydroxylase (TH) antibody, which stains catecholamine-producing cells in the medulla [8]. We found that cOpn5L2 IR cells overlapped with TH-positive cells (Table S1), showing that cOpn5L2 is localized to the chromaffin body, which is the medullary component of the adrenal gland (Figure 3A–D, D′). High magnification of cOpn5L2 IR medullary cells revealed that cOpn5L2 protein is localized to the cytoplasm (Figure 3D–F).

### Localization of cOpn5L2 in the brain

**Figure 4:**
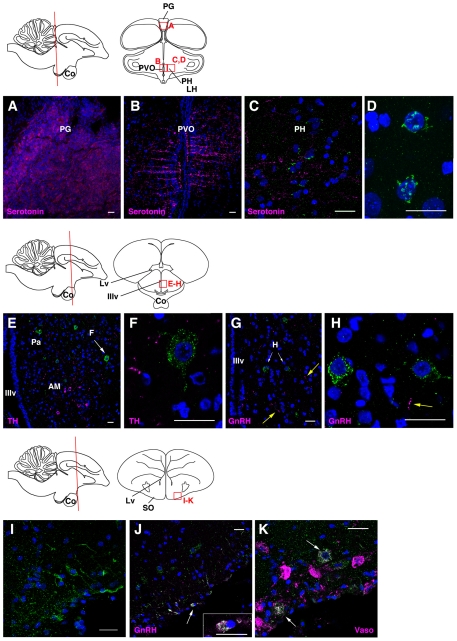
Distribution of cOpn5L2 immunoreactive neurons in chicken brain at P10. Three coronal levels, from posterior hypothalamus to preoptic regions, are illustrated. The approximate location of these regions is shown on the sagittal schema of chicken brain on the left. Red boxes indicate the areas of the confocal images. cOpn5L2 IR neurons and perikarya are indicated in green. The scale bars represent 20 µm. In all panels except (D, I), the sections were co-stained by two antibodies. The marker antibodies (magenta) used are indicated in each panel. For all images the DAPI stain is blue. ***A–D***, Coronal sections through the posterior hypothalamus. ***A***, Pineal gland (PG), in which there are no cOpn5L2 IR cells. As a positive control, PG was stained with an anti-serotonin antibody. ***B***, Paraventricular organ (PVO), in which there are no cOpn5L2 IR cells. As a control, the PVO was stained with an anti-serotonin antibody. ***C***, The posterior hypothalamic nucleus (PH), located laterally to the PVO. Weak but distinct immunoreactivity for cOpn5L2 is observed. ***D***, High magnification of cOpn5L2 IR cells in the lateral hypothalamic area (LH), shown in Figure 2F. In these cells, thread-like immunoreactive signals are seen. ***E–H***, Coronal sections through the anterior hypothalamus. ***E***, Paraventricular nucleus (Pa) and nucleus anterior medialis hypothalami (AM). cOpn5L2 IR cells are found in the Pa, dorsal to the TH IR cells in the AM. ***F***, High magnification of a cOpn5L2 IR cell shown in (E). The large soma is immunoreactive for cOpn5L2, and TH IR fibers are seen in the vicinity of the cOpn5L2 IR cell. ***G***, Paraventricular nucleus, in which cOpn5L2 IR cells and GnRH IR fibers (yellow arrows) are scattered. ***H***, High magnification of two cOpn5L2 IR cells shown in (G). A yellow arrow indicates a GnRH IR fiber. ***I–K***, Coronal sections through the preoptic region. ***I***, Supraoptic nucleus (SO), in which cOpn5L2 IR somata and long fibers are seen. The cOpn5L2 IR neuron is bipolar. ***J***, In the SO, cOpn5L2 IR and GnRH IR cells are observed, shown in white. High magnification of a representative cell (arrow) is shown in the inset. ***K***, In the SO, some vasotocin (vaso) IR cells are also positive for cOpn5L2 (arrows).

**Figure 5:**
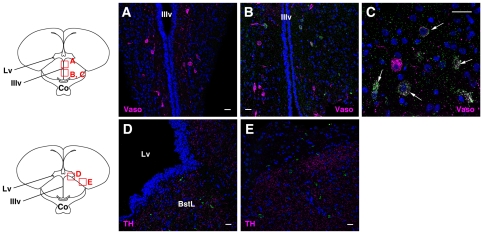
Distribution of cOpn5L2 immunoreactive neurons in the forebrain. ***A–C***, Coronal sections through the anterior hypothalamus at P10. ***A***, The dorsal part of the paraventricular nucleus, in which many vasotocin-positive cells are seen, but no cOpn5L2 IR cells are observed. ***B***, Paraventricular nucleus, ventral to the region shown in (A), in which cOpn5L2 IR cells are seen. ***C***, High magnification of paraventricular nucleus. A different section from that shown in (B). Some vasotocin IR cells are also positive for cOpn5L2 (arrows). ***D***, ***E***, Coronal sections through the anterior hypothalamus. Images are focused on the telencephalon. ***D***, cOpn5L2 IR cells are found in the bed nucleus of the stria terminalis, lateral part (BstL), in which TH IR fibers are prominent. ***E***, cOpn5L2 IR cells are also scattered in the lateral region of the telencephalon. Co, optic chiasm; IIIv, third ventricle; Lv, lateral ventricle. The scale bars represent 20 µm.

Non-mammalian species have been known to possess photoreceptors outside the eye, such as in the deep brain and pineal gland. We found that, in contrast to cOpn5m, cOpn5L2 was not present in the pineal gland (Figure 4A). Since deep brain photoreceptors of the avian species have been found in the ventral forebrain (reviewed in [9]), we focused on the forebrain to examine the localization of cOpn5L2. We first examined whether cOpn5L2 was localized to the paraventricular organ (PVO) of the hypothalamus. The PVO is composed of cerebrospinal fluid-contacting neurons, whose knob-like terminals protrude into the lumen of the third ventricle [10], where cOpn5m is co-localized with serotonin and likely regulates testicular growth in birds [5], [6]. We found that cOpn5L2 was not localized to the PVO (Figure 4B), but was present in a subset of cells located laterally to the PVO, the posterior hypothalamic nucleus, toward the lateral hypothalamic area (Figure 4C; Figure 2F, Figure 4D). In the anterior hypothalamus, more prominent cOpn5L2 IR was observed in a small subset of cells of the paraventricular nucleus, located just dorsal to the region where TH-positive cells are present (Figure 4E, F). It is known that gonadotropin-releasing hormone (GnRH) IR perikarya are located in the medial preoptic nucleus [11]. We found that cOpn5L2 IR cells were located ventral to the region where the GnRH IR cells reside (Figure 4G, H; Figure S1). cOpn5L2 was also localized to a subset of cells in the supraoptic nucleus (Figure 4I), where cOpn5L2 IR perikarya and fibers were partly immunoreactive to the anti-GnRH antibody (Figure 4J). We further examined whether cOpn5L2 IR cells overlapped with vasotocin IR cells in these regions. Vasotocin is a vasopressin-like nonapeptide in birds, which is produced by the hypothalamic neurosecretory magnocellular neurons of supraoptic and paraventricular nuclei (Figure 4K, Figure 5A) ([12]; reviewed in [13]). cOpn5L2 IR cells in the supraoptic and paraventricular nuclei partly overlapped with vasotocin IR cells (Figure 4K; Figure 5B, C) (Table S2). In contrast, cOpn5L2 IR somata were not observed in the dorsal part of the paraventricular nucleus where numerous vasotocin IR cells were found (Figure 5A).

In telencephalon, cOpn5L2 IR cells were found in the ventrolateral region to the lateral ventricle, which is the lateral part of the bed nucleus of stria terminalis (Figure 5D). This region corresponds to part of the avian limbic system. The cOpn5L2 IR cells were widely spread across the middle to lateral region of the forebrain (Figure 5E).

### Localization of cOpn5L2 in the retina

**Figure 6:**
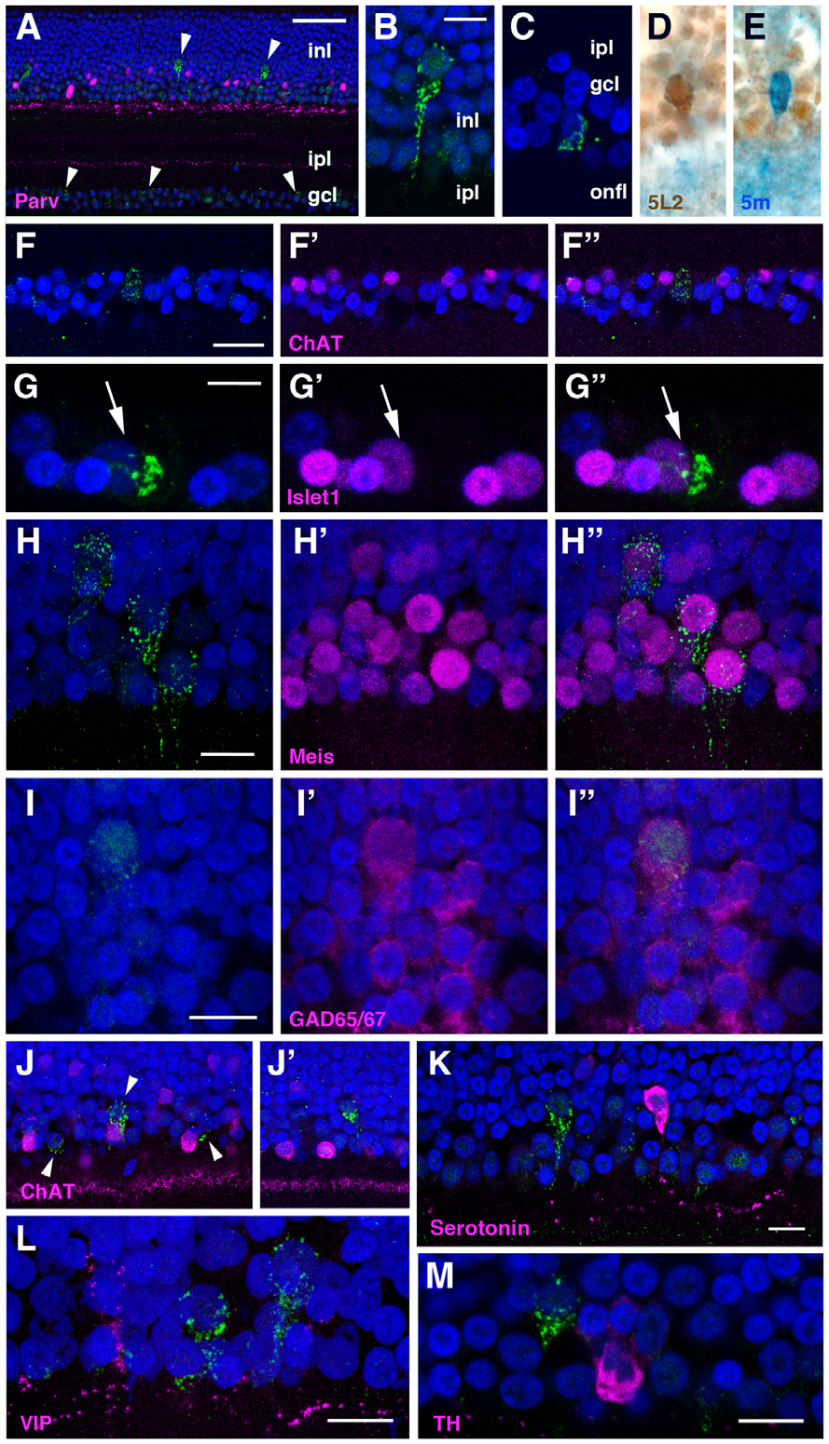
Immunohistochemistry of the chick retina at P10. ***A***, cOpn5L2 IR cells in the inner nuclear layer (inl) and ganglion cell layer (gcl) (green, arrowheads). Parvalbumin is visualized in magenta to reveal subsets of amacrine cells in the inner nuclear layer (inl) and sublamina I and V in the inner plexiform layer (ipl) [47]. ***B***, A representative cOpn5L2 IR cell in the INL. ***C***, A representative cOpn5L2 IR cell in the GCL. ***D***, A cOpn5L2 IR cell in the INL after two-color ABC immunostaining. ***E***, A cOpn5m IR cell in the INL of the same retinal section as shown in (D). ***F-F″***, The cOpn5L2 IR cell (green in ***F***, ***F″***) in the GCL is not positive for ChAT (magenta in F′, F″). ***G-G″***, The cOpn5L2 IR cell (arrow) in the GCL (G, G″) is positive for Islet1 (G′, G″). ***H-H″***, cOpn5L2 IR cells in the INL (H, H″) are positive for Meis (H′, H″). ***I-I″***, The cOpn5L2 IR cell in the INL (I, I″) is positive for GAD65/67 (I′, I″). ***J***, ***J′***, cOpn5L2 IR cells in the INL (green, arrowheads in J) are not positive for ChAT. Some cOpn5L2 IR cells are adjacent to ChAT IR cells (J), and others are separate from the ChAT IR cells (J′). ***K***, One cOpn5L2 IR cell in the vicinity of a serotonin IR cell, while the other is located apart from it. ***L***, cOpn5L2 IR cells are not positive for VIP. ***M***, A cOpn5L2 IR cell adjacent to a TH IR cell. For all images, DAPI is blue. Scale bars, 50 µm (A), 10 µm (B–E, G-I″, K–M), and 20 µm (F-F″, J, J′).

In the retina, cOpn5L2 protein was localized to a subset of cells of the ganglion cell layer (GCL) and the inner half of the inner nuclear layer (INL) (Figure 6A–C, Figure S2). Double staining using the two antibodies against cOpn5L2 and cOpn5m, a mammalian type Opn5 [5], [7], showed that cOpn5L2 IR cells in the INL were not positive for cOpn5m (Figure 6D, E). In contrast, we were not successful in detecting reliable signals for cOpn5L2 using the avidin biotin complex method in GCL cells, which precluded us from determining whether cOpn5L2 IR cells in the GCL overlapped with cOpn5m IR cells or not. We next sought to examine whether these cOpn5L2 IR cells were subsets of retinal ganglion cells or amacrine cells by using known retinal cell markers. In the GCL, displaced cholinergic amacrine cells are present whose somata are located adjacent to the inner plexiform layer [14]. Using antibodies to choline acetyltransferase (ChAT), we found that cOpn5L2 IR cells in the GCL were not stained by this antibody (Figure 6F-F″), indicating that they are not displaced cholinergic amacrine cells. Instead, the cOpn5L2 IR cells were positive for Islet1 (n = 15/20), a homeodomain-containing transcription factor expressed by most of the retinal ganglion cells [15] (Figure 6G-G″). Thus, the vast majority of cOpn5L2 IR cells in the GCL are likely a subset of retinal ganglion cells.

On the other hand, we found that cOpn5L2 IR cells in the INL express Meis, a subfamily of homeoproteins (Figure 6H-H″). Two Meis family members have been identified in the chicken genome [16]. Although the antibody used in this study cannot discriminate between the two Meis proteins, Meis2 is known to be expressed by a subpopulation of γ-aminobutyric acid (GABA) ergic amacrine cells in vertebrate mature retina, including near-hatched chick retina [17]. In agreement with these findings, cOpn5L2 IR cells in the INL were positive for glutamic acid decarboxylase (GAD) 65/67, which is a rate-limiting enzyme in the synthesis of GABA (Figure 6I-I″). In contrast, the cOpn5L2 IR cells in the INL were not positive for ChAT (Figure 6J, J′), serotonin (Figure 6K, Figure S3), vasoactive intestinal peptide (Figure 6L), or tyrosine hydroxylase, a marker for dopaminergic amacrine cells (Figure 6M). Thus, cOpn5L2 IR cells in the INL are most likely a subset of GABAergic amacrine cells.

## Discussion

In this study, we have characterized the molecular properties of another type of chicken Opn5, cOpn5L2. This pigment is a UV-sensitive bistable pigment that activates Gi-type G proteins in a state of having an all-*trans*-retinal chromophore. Immunohistochemical studies have revealed that cOpn5L2 protein is localized to subsets of cells in the retina and deep brain that are distinct from those for cOpn5m, and to the adrenal gland, which is not photoreceptive but an endocrine organ.

### cOpn5L2 is a functional GPCR in the Opn5 (neuropsin) group

We previously named Opn5L2 as Opn5-like 2 because the amino acid sequences are most closely related to Opn5, although their identities and similarities are relatively low, even in the core transmembrane region (34% identity/55% similarity between cOpn5m and cOpn5L2) [7]. Since this study showed that the molecular properties of cOpn5L2 are quite similar to that of cOpn5m, we concluded that cOpn5L2 functionally belongs to the Opn5 (neuropsin) group. We also showed that cOpn5L2 can activate G proteins, although it does not have the typical E/DRY motif conserved among the class A GPCRs or rhodopsin family. This triplet of amino acids is located at the boundary between helix III and intracellular loop 2 and is thought to be required for G protein activation ([18]; reviewed in [19], [20]). Specifically, tyrosine is the least conserved amino acid and is generally not important for receptor function (reviewed in [21]). Arginine is one of the most conserved residues in rhodopsin-like GPCRs and stabilizes both the inactive and the activated conformations of GPCRs [22], [23], [24]. Mutation in the glutamic acid/aspartic acid leads to a constitutive active state of rhodopsin family GPCRs [25], [26], [27]. Taken together, these studies indicate that cOpn5L2 may somewhat exhibit agonist-independent basal receptor activity, since it has an IRF motif instead of E/DRY and can activate G proteins.

### Comparison of the molecular properties between cOpn5L2 and cOpn5m

cOpn5L2 shares several molecular properties with cOpn5m, and both are sensitive to UV light and activate Gi-type G proteins. However, these proteins have differences in the characteristics of the visible light-absorbing forms. The absorption maximum of the form in cOpn5L2 (521 nm) is approximately 50 nm longer than that of cOpn5m (474 nm). Thus, the regeneration of the UV light-absorbing form is caused by irradiation with different wavelengths of visible light between cOpn5L2 and cOpn5m. In other words, cOpn5L2 can be inactivated by the absorption of longer wavelength light, which could more easily transmit into the head. Moreover, the efficiency of G protein activation by the visible light-absorbing form of cOpn5L2, which is produced by direct binding to exogenous all-*trans*-retinal, is comparable to that after UV light irradiation of the 11-*cis*-retinal bound form. However, this is not the case for cOpn5m: The activation efficiency by cOpn5m with direct binding to exogenous all-*trans*-retinal, is significantly lower than that after UV light irradiation of the 11-*cis*-retinal bound form [5]. Taken together, extraocular, deep brain cOpn5L2 more likely functions as a light-dependent inactivating pigment after direct binding to available all-*trans*-retinal without the visual cycle mechanism that provides a supply of 11-*cis* retinal.

### cOpn5L2 in the adrenal gland: cOpn5L2 may act as a chemosensor?

Although a previous study showed that gonad-adrenal activity of the quail is increased in long day conditions through the vasotocinergic system of the brain [28], there have been no reports on the direct photosensitivity of the adrenal glands to date. Therefore, it is hard to imagine that UV to visible light can be transferred to such a deep internal organ. In this regard, it is worth considering that cOpn5L2 can bind to all-*trans*-retinal and activate G protein even before irradiation (Figure 1E, G), which is not seen for classical bleaching photopigments, such as rod and cone opsins. Therefore, we hypothesize that under specified circumstances, cOpn5L2 might be able to bind to all-*trans*-retinal as a ligand unrelated to light absorption. Recently, it was reported that a *Drosophila* rhodopsin, Rh1, is not only involved in light sensation but also in temperature discrimination [29]. The postulated scenario is that a still-unidentified accessory factor that interacts with this rhodopsin accelerates its intrinsic thermal activity. Furthermore, the activity as thermosensor can be replaced by other *Drosophila* rhodopsins as well as mammalian melanopsin. Thus, it is conceivable to assume that cOpn5L2 acts as a chemosensor that detects certain chemical stimuli such as all-*trans*-retinal, although this hypothesis needs to be determined in future studies.

Naturally produced catecholamines include norepinephrine, epinephrine, and dopamine, but the main secretory products of the adrenal medulla are norepinephrine and epinephrine [30]. These adrenal catecholamines have diverse functions that affect the vascular system, carbohydrate metabolism, and neural activities. This study showed that cOpn5L2 is localized to the adrenal medulla that produces catecholamines, suggesting the possibility that GPCR signaling through cOpn5L2 modulates the secretion or biosynthesis of the adrenal catecholamines or vice versa.

### cOpn5L2 in the brain: cOpn5L2 signaling modulates functions of reproductive neuropeptides?

This study has elucidated the localization of cOpn5L2 in the post-hatch chick brain. Its distribution is different from that of cOpn5m: cOpn5L2 is localized to several nuclei in the anterior hypothalamus, but is not present in the pineal gland or PVO of the hypothalamus. Characteristically, cOpn5L2 IR cells partly overlapped with vasotocin IR or GnRH IR cells in the anterior hypothalamus. It is known that many species of birds are photoperiodic, whereby long days have a stimulatory effect on the reproductive activity (reviewed in [31]). The sensory receptor that mediates the response to photoperiod is thought to be located in the brain (reviewed in [32]). On the other hand, immunohistochemical studies have shown that the GnRH-system is influenced by photoperiod and mirrors sexual differentiation in the quail brain [33]. Thus, UV photoreception through cOpn5L2 in these hypothalamic nuclei may regulate reproductive activities through these neuropeptides. It is worth mentioning that cOpn5L2 IR somata are located in the deep brain nuclei more than 100 µm laterally from the third ventricle (Figure 4E), while cOpn5m IR cells are located in the wall of the third ventricle [5], [6]. Given that cOpn5L2 has a basal activation ability when it couples to all-*trans*-retinal in the dark state, it is also crucial to inactivate cOpn5L2-GPCR signaling through visible light irradiation in these brain nuclei.

A recent study has shown that testicular growth is induced by short-wave photo-stimulation in eye-patched, pinealectomized quail, and that the quail Opn5m IR neurons from the PVO seem to project to the external zone of the median eminence [6]. They suggested that testicular growth through Opn5m is mediated by the induction of thyroid stimulating hormone expression in the pars tuberalis of the posterior hypothalamus and secretion of GnRH from the nerve termini. However, the possible action of cOpn5L2 seems to be totally different: cOpn5L2 exists in subsets of vasotocinergic or GnRHergic neurons of paraventricular and supraoptic nuclei, which are known to regulate reproductive activity. That is, these neuroendocrine cells include cOpn5L2-expressing photo- or chemoreceptor cells in birds. Vasotocin, which was originally characterized as the antidiuretic hormone, also shows vasomotor and thermoregulatory effects and can even control oviposition (reviewed in [13] [34]). Thus, cOpn5L2-GPCR signaling might be involved in regulating these functions as well.

### cOpn5L2 in the retina: A subset of GABAergic amacrine cells possesses the cOpn5L2 photopigment

cOpn5L2 protein is localized to subsets of cells in the GCL and INL, which is a similar characteristic to cOpn5m. However, double immunostaining showed that cOpn5L2 IR amacrine cells are a different subset from those of cOpn5m in the retina, although this study could not determine whether this was also true for GCL cells. The current study and previous studies have shown that cOpn5L2 IR amacrine cells and *cOpn5m*-expressing amacrine cells are GABAergic [7]. It is known that retinal GABA levels show a circadian rhythm in rodents [35], and the in vivo administration of GABA agonists modulates both dopamine and melatonin synthesis in the chicken retina [36]. In addition to the pineal gland, the retina itself displays many rhythmic physiological events, such as melatonin and dopamine production and secretion [37]. Although melanopsin is postulated to be responsible for the rhythmic synthesis of these bioactive substances, the two Opn5 cognates, cOpn5L2 and cOpn5m, may also play a similar role in the chick retina though the action of the inhibitory neurotransmitter GABA.

### Conclusions

**Figure 7:**
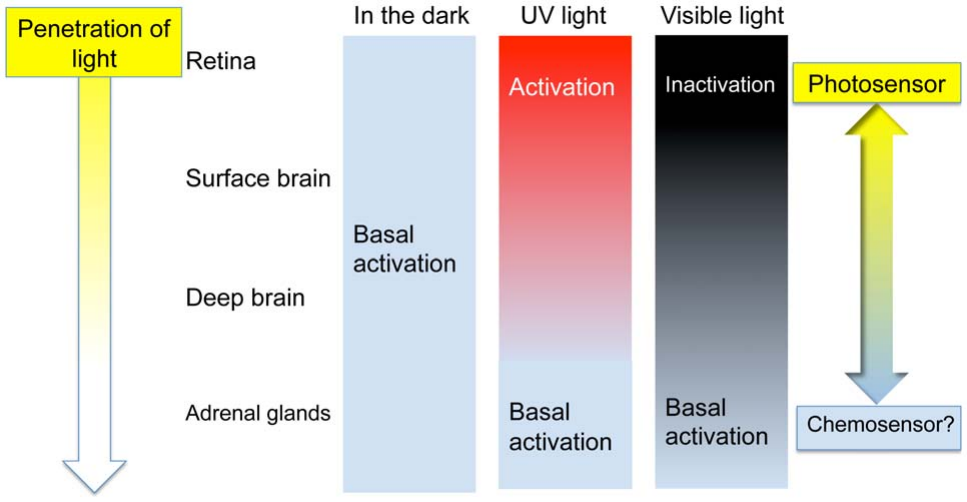
Functions of cOpn5L2 may depend on how much light is available to the cOpn5L2-expressing tissues. Availability of retinoids and interactions with cOpn5L2 may also influence whether photons or ligands transduce signals.

We have for the first time characterized the molecular properties of cOpn5L2, a second Opn5-like photopigment in birds. cOpn5L2 exhibits UV-sensitive GPCR activity in vitro, similar to the mammalian type Opn5, cOpn5m. However, its tissue distribution diverges from that of cOpn5m. Compared to cOpn5m, cOpn5L2 is localized to a different subset of GABAergic amacrine cells, including subsets of vasotocin or GnRH IR cells in the anterior hypothalamus, suggesting that cOpn5L2 has different functions than cOpn5m. Localization of cOpn5L2 in the brain, which is located laterally to the midline third ventricle, and in the adrenal gland, which is located deeply inside the body, suggests another physiological role of cOpn5L2 that is unrelated to photoreception. We therefore propose that cOpn5L2 exhibits dual mode of functional expression depending upon how much light is available to the tissues where cOpn5L2 is present (Figure 7). This rather nascent opsin, opsin 5-like 2, exclusively found in non-mammalian vertebrates, may dually function as a photosensor and a chemosensor, whose endogenous ligand and possible accessory proteins or retinoid cycle metabolism that modulate the ligand-binding affinity or accelerate the intrinsic chemosensor activity should be identified in the future studies.

## Materials and Methods

### Animals and ethics statement

Fertilized chicken eggs (*Gallus gallus domesticus*) were purchased from a commercial farm (Goto-furanjyo, Inc., Gifu, Japan; http://www.gotonohiyoko.co.jp/) and incubated at 37.5°C in a humidified incubator until hatching. Post-hatch chicks were housed under a 12∶12 light-dark cycle with food and water *ad libitum*. Animals were anesthetized and euthanized at Zeitgeber time 6–10. The brain and other tissues were dissected and processed for RNA extraction, western blotting, and immunohistochemistry. The use of animals in these experiments was in accordance with the guidelines established by the Ministry of Education, Culture, Sports, Science, and Technology, Japan, and University of Tokushima. The protocol was approved by the Committee on the Ethics of Animal Experiments of the University of Tokushima (Permit Number: 08089). All surgery as performed under anesthesia, and all efforts were made to minimize suffering.

### Preparation of purified cOpn5L2 pigments

The RNA fraction extracted from the chick retina (post-hatching day 10) was used for oligo (dT) primed synthesis of the cDNA, which was applied to 5′- and 3′ rapid amplification of cDNA (RACE). According to the sequences obtained by 5′- and 3′-RACE, the entire coding sequence of cOpn5L2 was amplified using primers as follows: cO5L2-S2, 5′- TTAAAACCTTCACCTGCTTTCACAGG-3′ and cO5L2-AS2, 5′- GCGACTCAGAATCTTGGCGTATGTT-3′. The nucleotide sequence has been submitted to the DDBJ database under the accession number DDBJ: AB368183. The cDNA of cOpn5L2 was tagged by the epitope sequence of the anti-bovine rhodopsin monoclonal antibody Rho1D4 (ETSQVAPA) at the C-terminus and was inserted into a mammalian expression vector pCAGGS [38]. The plasmid DNA was transfected into HEK293 cells using the calcium phosphate method. After 1 day of incubation, 11-*cis*- or all-*trans*-retinal was added into the medium (final retinal concentration, 5 µM). After additional incubation for 1 day in the dark, the cells were collected. The pigments were extracted with 1% dodecylmaltoside (DM) in buffer A (50 mM 4-[2-hydroxyethyl]-1-piperazineethanesulfonic acid [HEPES] [pH 6.5] and 140 mM NaCl) and were purified by Rho1D4-conjugated agarose. The purified pigments were eluted with 0.02% DM in buffer A containing the synthetic peptide that corresponded to the C-terminus of bovine rhodopsin [5].

### Preparation of G proteins

The rat Giα1 subunit was expressed in the *Escherichia coli* strain BL21 by using Giα1 cDNA constructed into the pQE6 plasmid vector and was purified as previously described [39]. The purified Giα1 was mixed with an equal amount of Gtβγ purified from bovine rod outer segments.

### Spectrophotometry and high performance liquid chromatography (HPLC) analysis

Absorption spectra were recorded at 0°C with a Shimadzu UV-2400 spectrophotometer. The sample was irradiated with UV light through a UV-D35 glass filter (Asahi Technoglass) or with yellow light through a Y-52 cutoff filter (Toshiba) from a 1 kW halogen lamp (Master HILUX-HR; Rikagaku). The absorption spectra of visible and UV light-absorbing forms of cOpn5L2 were calculated from the methods previously described [5]. Briefly, the spectral region at wavelengths longer than the maximum of the main peak of the spectrum difference between the visible and UV light-absorbing forms of the pigment was best-fitted with a template spectrum previously described [40], [41]. The best-fitting spectrum was considered to be the visible light-absorbing form of the pigment. The absorption spectrum of the UV light-absorbing form was then calculated by adding the visible light-absorbing form to the difference spectrum. The retinal configurations of each sample were analyzed by HPLC (LC-10AT VP; Shimadzu) equipped with a silica column (150×6 mm, A-012-3; YMC) according to the previous study [42].

### G protein activation assay

A radionucleotide filter-binding assay, which measures guanosine diphosphate (GDP)/guanosine triphosphate (GTP)γS exchange by G protein, was performed as previously described [5]. All procedures were carried out at 0°C. The assay mixture consisted of 50 mM HEPES (pH 7.0), 140 mM NaCl, 5 mM MgCl_2_, 1 mM dithiothreitol, 0.01% DM, 1 µM [^35^S] GTPγS, and 2 µM GDP. The purified cOpn5L2 (final concentration: 5 nM) was mixed with the G protein solution (final concentration: 600 nM) and was kept in the dark or irradiated with UV light for 1 min or with subsequent yellow light (>500 nm) for 1 min. After irradiation, the GDP/GTPγS exchange reaction was initiated by the addition of [^35^S] GTPγS solution into the mixture of the pigment and G protein. After incubation for the selected time in the dark, an aliquot (20 µl) was removed from the sample into 200 µl of stop solution (20 mM Tris-HCl [pH 7.4], 100 mM NaCl, 25 mM MgCl_2_, 1 µM GTPγS, and 2 µM GDP) and immediately filtered through a nitrocellulose membrane to trap [^35^S] GTPγS bound to G proteins. The amount of bound [^35^S] GTPγS was quantified by assaying the membrane with a liquid scintillation counter (Tri-Carb 2910 TR; PerkinElmer).

### Quantitative PCR

A panel of post-hatching (2 weeks) chick tissues (kidney, liver, heart, testis, lung, brain, muscle, spleen, retina, and adrenal glands) was dissected, snap frozen by liquid nitrogen, and stored at −80°C until required. The methods of RNA extraction, cDNA synthesis, PCR primers and conditions, and relative quantification of transcript levels were previously described [7]. The amplified cDNA fragments were confirmed by electrophoresis and sequencing to identify the desired ones.

### Antibodies

Specific antibodies were raised to the N-terminus or C-terminus of cOpn5L2 using the guinea pig. Each polyclonal antibody was raised against a 15 or 17 amino acid synthetic peptide conjugated to Keyhole Limpet Hemocyanin by Tanpaku-Seisei-Kogyo (Gunma, Japan), according to their standard procedures (N-term: MEEQYISKLHPVVDY; C-term: IRLSPTAKVESQGAARH). These antibodies were affinity purified prior to use by Tanpaku-Seisei-Kogyo. The primary antibodies used in this study included: mouse anti-tyrosine hydroxylase (TH) antibody (MAB318, Chemicon Millipore), mouse anti-Islet1 antibody (40.2D6, Developmental Studies Hybridoma Bank), rabbit anti-choline acetyltransferase (ChAT) antibody (#2017, a gift from Dr. Miles Epstein, University of Wisconsin, USA), rabbit anti-Meis antibody (MAB1614, Millipore), rabbit anti-glutamic acid decarboxylase (GAD) 65/67 antibody (ab11070, Abcam), rabbit anti-vasoactive intestinal peptide (VIP) antibody (AR443-5R, Biogenex), rabbit anti-serotonin antibody (a gift from Dr. David V. Pow, University of Newcastle, Australia), rabbit anti-gonadotropin releasing hormone (GnRH) antibody (ab5617, Abcam), and rabbit anti-vasopressin antibody (T-4563, Bachem). The secondary antibodies used in this study included: Cyanine 3 (Cy3)-labeled donkey anti-mouse and anti-rabbit IgG (Jackson Immunoresearch, catalogue numbers 715-166-150 and 715-166-152, respectively), and Alexa Fluor 488-labeled goat anti-guinea pig IgG (Invitrogen, A-11073).

### Western blotting

The extract from the cOpn5L2-transfected HEK293 cells was subjected to 12% sodium dodecyl sulfate-polyacrylamide gel electrophoresis (SDS-PAGE), transferred onto a polyvinylidene difluoride (PVDF) membrane, and probed with anti-cOpn5L2-C-term (diluted 1∶1000) or anti-cOpn5L2-N-term (diluted 1∶1000) antibody. Immunoreactive proteins were detected by the avidin biotin complex (ABC) method and visualized with a horseradish peroxidase-diaminobenzidine (DAB) reaction.

### Fixation and sectioning

Chick eye, brain, and adrenal glands were quickly dissected, fixed with 4% paraformaldehyde in phosphate-buffered saline (PBS) for 3 h (for the eyes) or 16 h (for brain and adrenal glands) at 4°C and then transferred to 20% sucrose until they sank. After embedding the samples in optimal cutting temperature compound (Sakura, Japan), the tissues were sectioned with a cryostat (Leica) at a 20 µm thickness. Sections were thaw-mounted onto SuperFrost Plus slides (Fisher Scientific), dried at 37°C, and stored at −30°C until use. The anatomy of the chick brain was determined according to Puelles et al. (2007) [43] and the nomenclature adopted in this study was based on this atlas and previous studies [44], [45], [46].

### Immunohistochemistry

Fluorescent immunolabeling was performed using standard techniques. Briefly, all slides were blocked for 30 min at room temperature in PBS Triton X-100 (0.25%) (PBST) with 5% serum from the same species as the corresponding secondary antibodies. Primary antibodies were diluted in PBST with 5% serum and secondary antibodies in PBS. All wash steps were performed 3 times with PBST for 5 min each. Primary antibodies (anti-cOpn5L2-C-term [diluted 1∶2000–1∶500], anti-cOpn5L2-N-term [diluted 1∶500], anti-TH [diluted 1∶250], anti-Islet1 [diluted 1∶50], anti-ChAT [diluted 1∶1000], anti-Meis [diluted 1∶500], anti-GAD65/67 [diluted 1∶200], anti-serotonin [diluted 1∶2000], anti-VIP [diluted 1∶1], anti-GnRH [diluted 1∶1000], and anti-vasopressin [diluted 1∶500]) were incubated for 5 h at room temperature or for 16 h at 4°C. The anti-vasopressin antibody employed here has 100% cross-reactivity to avian vasotocin (Arginine8-vasotocin) according to the manufacturer's instructions. Secondary antibodies were incubated for 1.5 h at room temperature and diluted 1∶750. For double fluorescent labeling experiments, the slides were incubated with primary antibodies and secondary antibodies in a sequential manner: anti-cOpn5L2-C-ter followed by anti-TH or other antibodies. The cell nuclei were stained with 4′,6-diamidino-2-phenylindole (DAPI) (Vector Laboratories) or DRAQ5™ (Biostatus) and filamentous actin was stained with rhodamine-phalloidin (Invitrogen, R415). The slides were mounted with anti-fade mountant, Vectashield (Vector, H-1500 or H-1400). Fluorescent images were collected using a Leica TCS-SP5 confocal laser-scanning microscope, excitation 405, 488, 543, and 633 nm with emission wavelengths of 424–489, 505–539, 551–618, and 679–702 nm for DAPI, green, Cy3, and DRAQ5, respectively.

### Double immunostaining of cOpn5L2 and cOpn5m

Since both of these antibodies were made in guinea pig, retinal sections were first immunostained with anti-cOpn5L2(C) antibody [5] using Vectastain ABC Elite Kit and ImmPACT™ DAB Peroxidase Substrate (Vector), and subsequently treated with citrate acid solution (pH 6) at 121°C for 15 minutes. The samples were then sequentially stained with anti-cOpn5m antibody using the alkaline phosphatase (AP) system (Vectastain ABC-AP Kit and Vector Blue Alkaline Phosphatase Kit, Vector). We could not achieve reliable detection of cOpn5L2 immunoreactive signals in the retinal ganglion cell layer using the ABC method.

### Colocalization analysis

We assessed numbers for the percentage of overlap of cOpn5L2 and marker proteins within cells using the ImageJ software plug-in *Colocalisation Thresholds* (http://rsbweb.nih.gov/ij/). Briefly, after sequential immunostaining of both orders (anti-cOpn5L2 to anti-TH, or anti-TH to anti-cOpn5L2), five confocal images were taken for each staining order and analyzed for colocalization (Table S1). The images were taken under the same confocal microscopic conditions and contained 229±59 cells (mean±standard deviation [S. D.], n = 10) of the adrenal gland. Proportions of overlapping cells in the paraventricular and supraoptic nuclei, and retinal ganglion cells were calculated by counting cells manually on confocal images with a ×63 objective zoom 2 (Table S2 and in the text).

## Supporting Information

Table S1 **Proportion of overlapping cells in the adrenal gland as revealed by Manders' coefficient (M1, M2) using a colocalization tool (see** **Materials and Methods** **).** *M1, proportion of cOpn5L2 immunoreactive (IR) cells among tyrosine hydroxylase (TH)-positive cells. **M2, proportion of TH-positive cells among cOpn5L2 IR cells.
(TIF)

Table S2 **Numbers of overlapping cells in the brain nuclei.**
(TIF)

Figure S1 **A GnRH immunoreactive cell, locating dorsal to the region where cOpn5L2 immunoreactive cells reside.**
(TIF)

Figure S2 **An enlarged image of** **Figure 6A** **.** See legend for Fig. 6A.
(TIF)

Figure S3 **A full image of** **Figure 6K** **.** Serotonin immunoreactivity is localized to an amacrine cell body in the inner nuclear layer (INL), and two synaptic strata in the inner plexiform layer (IPL). A weakly immunoreactive bipolar cell is also localized to the INL (arrow), as reported previously (George A et al. (2005) Exp. Eye Res. 81, 616–625).
(TIF)
