# Supplementary figures and images for: A Non-Mammalian Type Opsin 5 Functions Dually in the Photoreceptive and Non-Photoreceptive Organs of Birds
Source: PLoS One. 2012 Feb 14;7(2):e31534. doi: 10.1371/journal.pone.0031534 (PMC3279408; doi:10.1371/journal.pone.0031534)

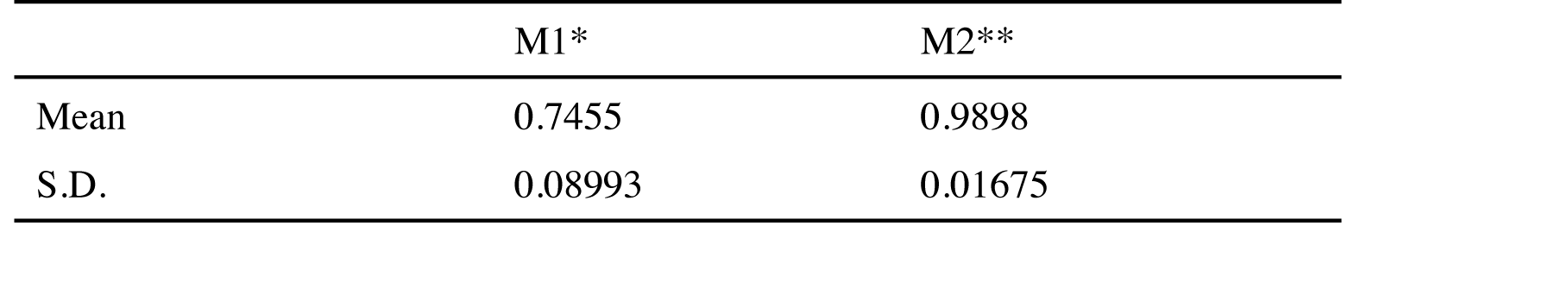

Supplement: Table S1 — Proportion of overlapping cells in the adrenal gland as revealed by Manders' coefficient (M1, M2) using a colocalization tool (see Materials and Methods ). *M1, proportion of cOpn5L2 immunoreactive (IR) cells among tyrosine hydroxylase (TH)-positive cells. **M2, proportion of TH-positive cells among cOpn5L2 IR cells. (TIF) [file pone.0031534.s001.tif]

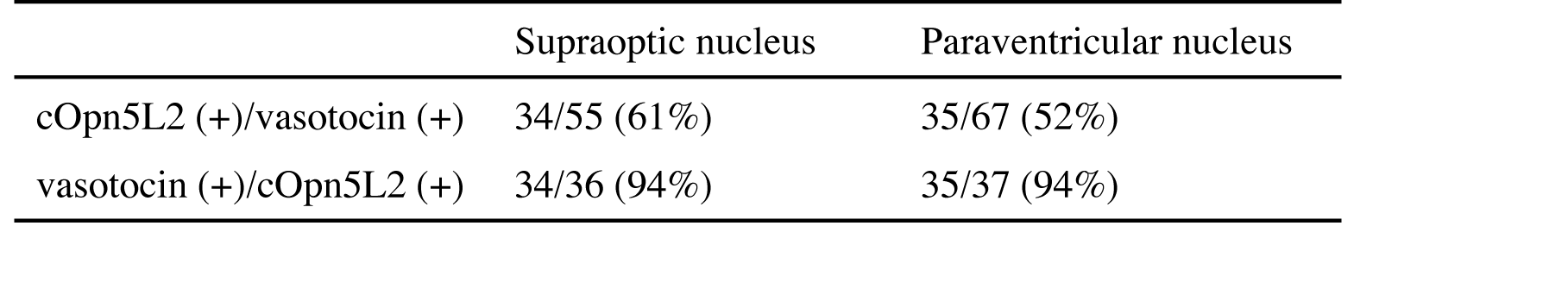

Supplement: Table S2 — Numbers of overlapping cells in the brain nuclei. (TIF) [file pone.0031534.s002.tif]

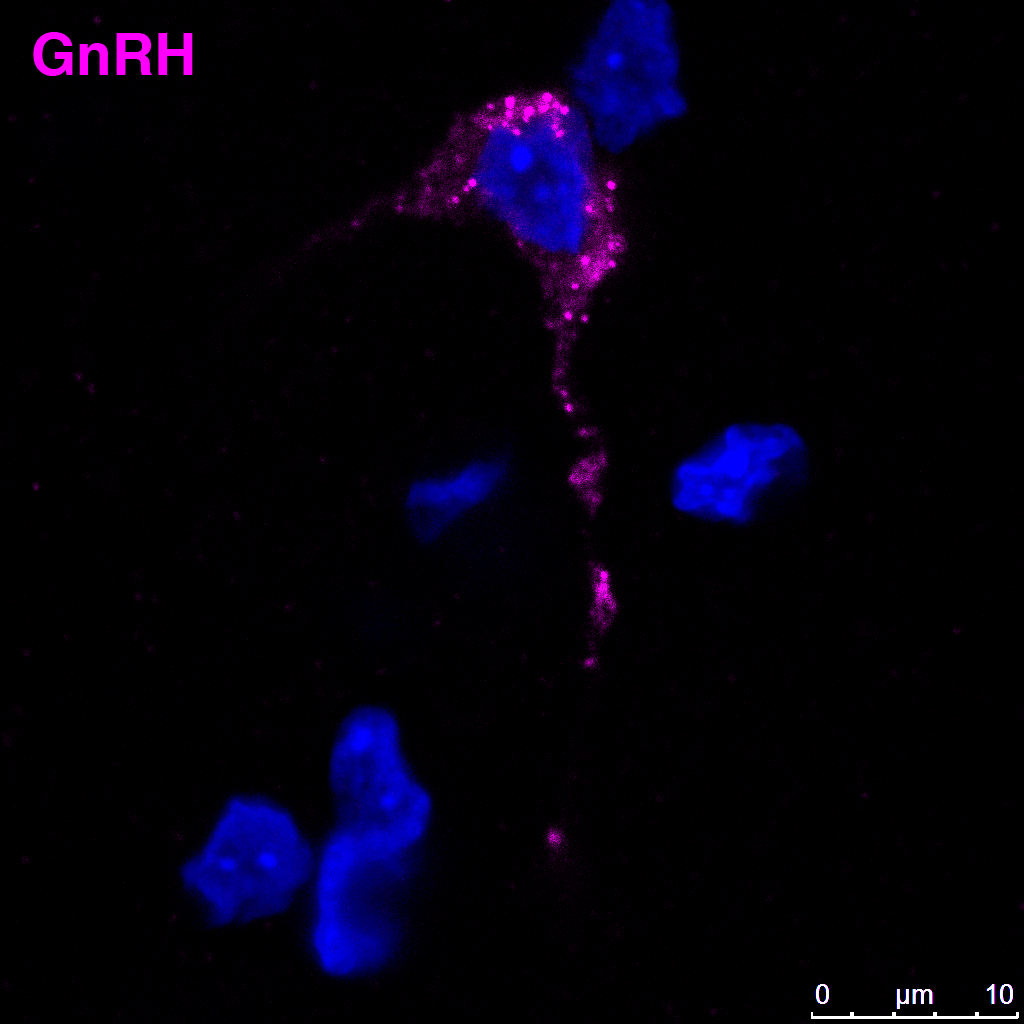

Supplement: Figure S1 — A GnRH immunoreactive cell, locating dorsal to the region where cOpn5L2 immunoreactive cells reside. (TIF) [file pone.0031534.s003.tif]

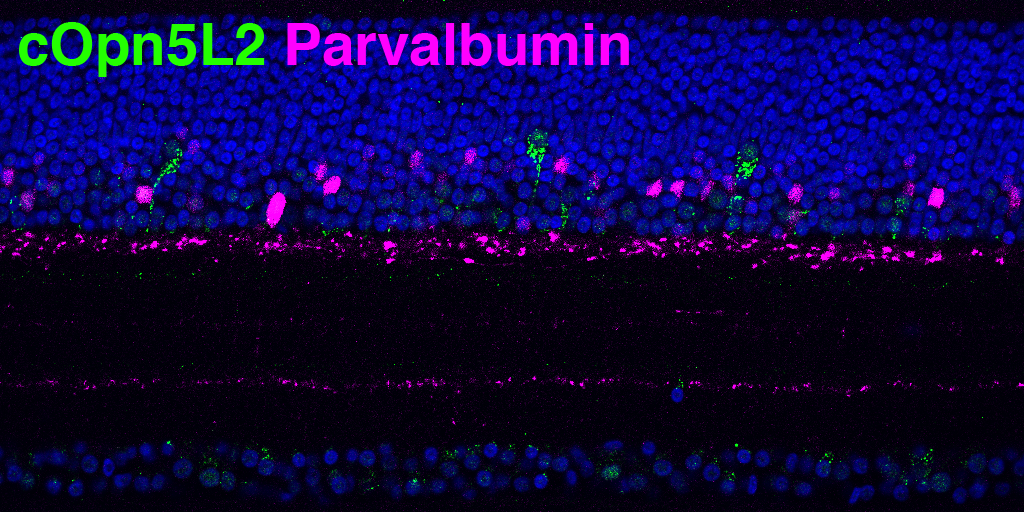

Supplement: Figure S2 — An enlarged image of Figure 6A . See legend for Fig. 6A. (TIF) [file pone.0031534.s004.tif]

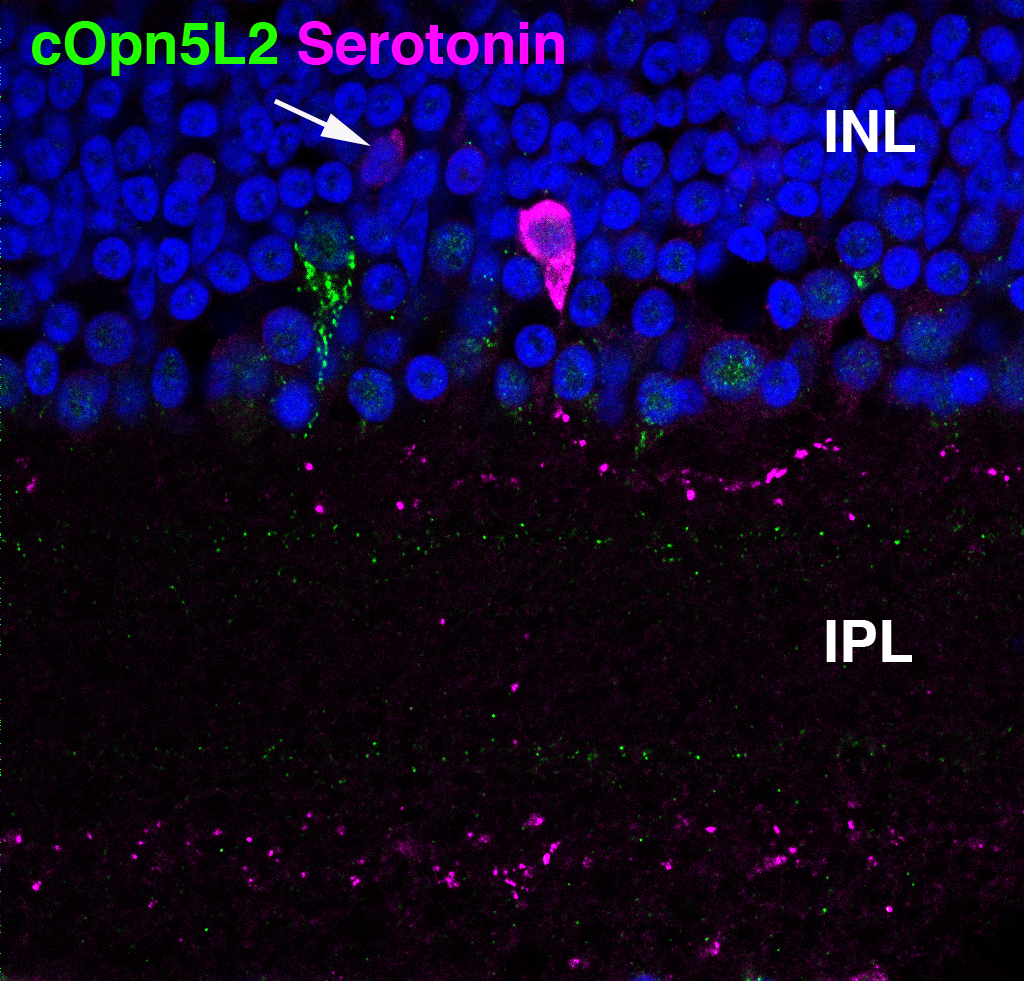

Supplement: Figure S3 — A full image of Figure 6K . Serotonin immunoreactivity is localized to an amacrine cell body in the inner nuclear layer (INL), and two synaptic strata in the inner plexiform layer (IPL). A weakly immunoreactive bipolar cell is also localized to the INL (arrow), as reported previously (George A et al. (2005) Exp. Eye Res. 81, 616–625). (TIF) [file pone.0031534.s005.tif]
